# The Effect of Three or Six Years of Denosumab Exposure in Women With Postmenopausal Osteoporosis: Results From the FREEDOM Extension

**DOI:** 10.1210/jc.2013-1597

**Published:** 2013-08-26

**Authors:** Henry G. Bone, Roland Chapurlat, Maria-Luisa Brandi, Jacques P. Brown, Edward Czerwiński, Marc-Antoine Krieg, Dan Mellström, Sebastião C. Radominski, Jean-Yves Reginster, Heinrich Resch, Jose A. Román Ivorra, Christian Roux, Eric Vittinghoff, Nadia S. Daizadeh, Andrea Wang, Michelle N. Bradley, Nathalie Franchimont, Michelle L. Geller, Rachel B. Wagman, Steven R. Cummings, Socrates Papapoulos

**Affiliations:** Michigan Bone and Mineral Clinic (H.G.B.), Detroit, Michigan 48236; Institut National de la Santé et de la Recherche Médicale Unité Mixte de Recherche 1033 and Université de Lyon (R.C.), 69437 Lyon, France; University of Florence (M.-L.B.), 50139 Florence, Italy; Centre de Recherche du Centre Hospitalier Universitaire de Québec (J.P.B.) and Department of Medicine (J.P.B.), Faculty of Medicine, Laval University, Québec City, Québec, Canada G1V 4G2; Krakow Medical Center (E.C.), 31-501 Krakow, Poland; University Hospital of Lausanne (M.-A.K.), 1011 Lausanne, Switzerland; Sahlgrenska University Hospital (D.M.), 411 32 Göteborg, Sweden; Universidade Federal do Paraná (S.C.R.), 80060 Curitiba, Brazil; University of Liège (J.-Y.R.), 4020 Liège, Belgium; St Vincent Hospital (H.R.), 1060 Vienna, Austria; Hospital Universitario La Fe (J.A.R.I.), 46026 Valencia, Spain; Paris Descartes University (C.R.), 75014 Paris, France; University of California, San Francisco (E.V.), San Francisco, California 94107; Amgen Inc (N.S.D., A.W., M.N.B., N.F., M.L.G., R.B.W.), Thousand Oaks, California 91320; San Francisco Coordinating Center, California Pacific Medical Center Research Institute, and UCSF (S.R.C.), San Francisco, California 94107; and Leiden University Medical Center (S.P.), 2333 RC Leiden, The Netherlands

## Abstract

**Context:**

The Fracture Reduction Evaluation of Denosumab in Osteoporosis Every 6 Months (FREEDOM) extension is evaluating the long-term efficacy and safety of denosumab for up to 10 years.

**Objective:**

The objective of the study was to report results from the first 3 years of the extension, representing up to 6 years of denosumab exposure.

**Design, Setting, and Participants:**

This was a multicenter, international, open-label study of 4550 women.

**Intervention:**

Women from the FREEDOM denosumab group received 3 more years of denosumab for a total of 6 years (long-term) and women from the FREEDOM placebo group received 3 years of denosumab (crossover).

**Main Outcome Measures:**

Bone turnover markers (BTMs), bone mineral density (BMD), fracture, and safety data are reported.

**Results:**

Reductions in BTMs were maintained (long-term) or achieved rapidly (crossover) after denosumab administration. In the long-term group, BMD further increased for cumulative 6-year gains of 15.2% (lumbar spine) and 7.5% (total hip). During the first 3 years of denosumab treatment, the crossover group had significant gains in lumbar spine (9.4%) and total hip (4.8%) BMD, similar to the long-term group during the 3-year FREEDOM trial. In the long-term group, fracture incidences remained low and below the rates projected for a virtual placebo cohort. In the crossover group, 3-year incidences of new vertebral and nonvertebral fractures were similar to those of the FREEDOM denosumab group. Incidence rates of adverse events did not increase over time. Six participants had events of osteonecrosis of the jaw confirmed by adjudication. One participant had a fracture adjudicated as consistent with atypical femoral fracture.

**Conclusion:**

Denosumab treatment for 6 years remained well tolerated, maintained reduced bone turnover, and continued to increase BMD. Fracture incidence remained low.

Receptor activator of nuclear factor-κB ligand (RANKL) plays an essential role in mediating bone resorption through osteoclast formation, function, and survival (1, 2). After menopause, increased RANKL results in increased bone resorption and bone loss, which can lead to osteoporosis (3), a condition characterized by compromised bone strength and increased risk of fracture (4, 5).

Denosumab is a fully human monoclonal antibody that binds with high specificity to human RANKL (6, 7), thereby reducing osteoclast number and activity and decreasing bone resorption. In postmenopausal women with osteoporosis, denosumab significantly reduced bone turnover markers (BTMs), increased bone mineral density (BMD), and reduced new vertebral (68%), hip (40%), and nonvertebral (20%) fractures compared with placebo during the pivotal 3-year Fracture Reduction Evaluation of Denosumab in Osteoporosis Every 6 Months (FREEDOM) trial (8).

Evaluating the long-term safety and efficacy of denosumab is important because osteoporosis is a chronic disease requiring long-term treatment. A phase 2 dose-ranging study demonstrated that up to 8 years of continued denosumab treatment in a small group of women was well tolerated and associated with continued gains in BMD and maintained reductions in BTMs (9). There is increasing interest in the long-term effects of antiosteoporotic treatments, and it is important to confirm key clinical trial results. Therefore, in addition to the long-term phase 2 extension, the 3-year, phase 3 FREEDOM trial has been extended for 7 additional years, during which all participants receive open-label denosumab. We report here the effects of denosumab on BTMs, BMD, safety, and fracture rates for the first 3 years of the extension. For women from the FREEDOM placebo group who enrolled in the extension, these data (as the crossover group) provide a unique opportunity for comparison with the original 3-year denosumab FREEDOM observations because these subjects have now received 3 years of denosumab exposure. In addition, for women from the FREEDOM denosumab group who enrolled in the extension, these data (as the long-term group) allow for evaluation of safety and efficacy beyond 5 years of treatment.

## Patients and Methods

### Study design

The FREEDOM pivotal trial design (clinicaltrials.gov: NCT00089791) and the extension design (clinicaltrials.gov: NCT00523341) have been described in previous publications (8, 10) and are summarized here. FREEDOM was a phase 3, multicenter, randomized, double-blind, placebo-controlled, 3-year study conducted at 214 centers globally. Postmenopausal women who enrolled had a lumbar spine or total hip BMD T-score less than −2.5 at either location and −4.0 or greater at both locations and were 60–90 years old. Participants were randomized to receive placebo or 60 mg denosumab (Prolia; Amgen Inc) sc every 6 months for 3 years and were instructed to take calcium (≥1 g) and vitamin D (≥400 IU) daily. All women who completed the FREEDOM trial [ie, completed their 3 y visit and did not discontinue the investigational product (IP)] and did not miss more than one dose of IP were eligible to enter the extension. The extension was originally planned for 2 years but was subsequently extended to 7 years. At the end of 2 years, participants consented again to continue for the additional 5 years. During the extension period, all participants are scheduled to receive denosumab 60 mg sc every 6 months (±1 mo) with daily calcium and vitamin D. The study is ongoing and annual/preplanned data analyses are performed as part of continuing pharmacovigilance. The data reported here include the first 3 years of the extension, representing up to 6 years of denosumab exposure for the long-term group.

Representatives of the sponsor designed the study in collaboration with investigators and conducted the statistical analyses according to a prespecified plan. The study protocol was approved by an institutional review board or ethics committee for each site. Participants provided written informed consent. All authors had access to the data, participated in drafting or revising the manuscript, approved the final version for submission, and take responsibility for the integrity of the analysis.

### Study procedures

During the extension, study visits are scheduled at baseline (corresponding to the end of the FREEDOM trial) and every 6 months for 7 years.

Measurements of serum C-terminal telopeptide of type 1 collagen (CTX; Nordic Bioscience Diagnostics A/S) and procollagen type 1 N-terminal propeptide (P1NP; Orion Diagnostica Oy) were determined using overnight fasting serum samples collected from a subset of women who participated in the FREEDOM BTM substudy and continued in the extension. To increase the sample size of the BTM substudy, the protocol was amended to include additional participants at year 2 of the extension, and evaluation of these additional participants began at year 3 of the extension. Subsequent to the publication of the data from the first 2 years of the extension (10), additional samples for P1NP from the second year were identified in storage by the central laboratory. P1NP levels were measured in these samples and the data have been included in the current analysis. Undetectable values were imputed using the corresponding assay's established lower limit of detection value (CTX, 0.049 ng/mL; PINP, 10 μg/L).

BMD measurements were performed by dual-energy x-ray absorptiometry at the lumbar spine and proximal femur (all women) and at the forearm (subset of women) at extension baseline and years 1, 2, and 3.

Vertebral fractures were identified by a central facility (Synarc Inc) using the Genant semiquantitative grading scale (11) using thoracic and lumbar lateral radiographs obtained at extension baseline, year 2, and year 3. Prevalent vertebral fractures at baseline and new vertebral fractures were defined as described previously (10). Clinical and nonvertebral fractures required confirmation by a radiologist's report or diagnostic imaging and were documented as they occurred.

Subjects were queried about adverse events (AEs), clinical fracture information, and concomitant medications by study site staff every 3 months. Each potential case of osteonecrosis of the jaw (ONJ) was reviewed by an independent, blinded, external adjudication committee, as described previously (12).

An adjudication process was initiated during year 6 to review potential cases of atypical femoral fracture. Available x-ray images of femoral fractures that occurred at any time between baseline and year 3 of the extension were retrospectively reviewed by a panel at the central radiographic vendor site (Synarc Inc). Only the major criteria established by the ASBMR 2010 Task Force for Atypical Subtrochanteric and Diaphyseal Femoral Fractures (13) were used for adjudication by this panel: fracture location (subtrochanteric or femur diaphysis); fracture type (noncomminuted, simple transverse, or short oblique); no or minimal trauma; and the absence of any evidence of malignancy, prosthesis, pins, or other hardware at or near the fracture site prior to the fracture event. A finding of indeterminate was not permitted.

### Statistical analyses

The primary objective of the extension is to describe the safety and tolerability of denosumab. Secondary objectives include evaluation of the changes in BTMs and BMD and the incidence of vertebral and nonvertebral fractures.

Safety analyses included participants who received one or more doses of investigational product. Treatment groups were defined by the original randomized assignments in FREEDOM. For consistency with our prior report of the FREEDOM extension data (10), the Medical Dictionary for Regulatory Authorities version 13.0 was used to code and report AEs for this publication. The analyses of AEs included exposure-adjusted subject incidence rates and were descriptive. A selected group of serious AEs/AEs are reported, consistent with the primary FREEDOM publication (8) and the first long-term follow-up report (10).

Analyses of BTM included participants who received one or more doses of investigational product in the extension and had observed values at the time points of interest. Results are presented as actual values and correspond to medians and interquartile ranges. Analyses of BMD percentage change from baseline were conducted as described previously (10), required observed values at FREEDOM baseline and the time points of interest, and are presented as least squares means with 95% confidence intervals.

Crude subject incidence rates for new vertebral fracture and new and worsening vertebral fracture were calculated and Kaplan-Meier estimates of the cumulative incidence of clinical vertebral, all clinical, nonvertebral, major nonvertebral, and hip fractures were calculated. Nonvertebral fractures included low-trauma fragility fractures only, as defined previously (8). New vertebral and nonvertebral fracture incidence rates were also calculated for a per-protocol (PP) subset and a modified per-protocol (MPP) subset, both of which were prespecified. Both subsets included participants who were compliant with the protocol (characterized by the nonviolation of any of the important protocol deviations). The MPP subset further excluded subjects who missed two or more (not necessarily consecutive) doses of denosumab during the extension.

Because there was no placebo group in the extension, a simulation method developed for such an extension study design (14) was used to estimate expected fracture rates in a hypothetical cohort of placebo controls (virtual twin). As described previously, this modeled fracture risk for a theoretical placebo-treated population matched to actual study participants with regard to characteristics that were predictive of fracture risk in the participants in the original FREEDOM placebo group (10). Protocol-specified exploratory analyses were carried out comparing the model group's estimated lumbar spine and nonvertebral fracture rates with those observed in the treatment groups. Estimates of the relative risks (RRs) and their respective bootstrap 95% confidence intervals were calculated.

Fracture and safety data from all women enrolled in FREEDOM are summarized in this manuscript to allow for comparison with the 3-year extension results. For BTMs and BMD, data from women enrolled in the extension are presented.

## Results

**Figure 1.**
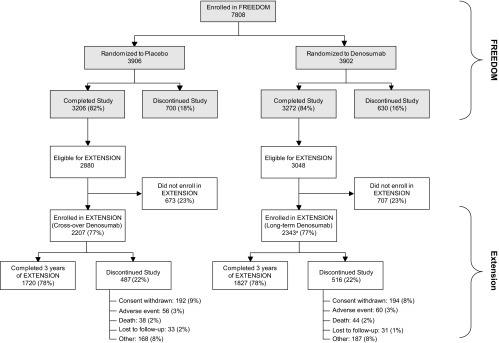
Disposition of all participants. All women who completed FREEDOM (ie, completed their 3 y visit, did not discontinue IP, and did not miss more than one dose) were eligible to participate in the FREEDOM extension. ^a^, Two women who discontinued denosumab also entered the extension in the long-term denosumab group.

There were 7808 participants enrolled in the FREEDOM pivotal trial (Figure 1). Of these, 5928 (76%) were eligible for enrollment in the extension and 4550 (2343 long-term, 2207 crossover) enrolled (77%). By the end of the third year, 78% (3547) of those enrolled remained on study. The proportion of participants who discontinued from the study overall and, for each reason, was similar between the long-term and crossover groups. The baseline characteristics for these groups at both the FREEDOM and extension baseline are shown in Table 1. Efficacy and safety data from the long-term and crossover groups are described separately below.

**Table 1.** Baseline Characteristics

	Long-Term Denosumab Extension Participants (n = 2343)		Crossover Denosumab Extension Participants (n = 2207)	
	FREEDOM Baseline	Extension Baseline	FREEDOM Baseline	Extension Baseline
Age, y	71.9 (5.0)	74.9 (5.0)	71.8 (5.1)	74.8 (5.1)
Age groups, n, %				
≥65	2209 (94.3)	2294 (97.9)	2067 (93.7)	2149 (97.4)
≥ 75	662 (28.3)	1258 (53.7)	624 (28.3)	1151 (52.2)
Years since menopause	23.7 (7.3)	26.7 (7.3)	23.7 (7.4)	26.7 (7.4)
Prevalent vertebral fractures, n, %	559 (23.9)	573 (24.5)	485 (22.0)	551 (25.0)
Prevalent nonvertebral fractures at age ≥55, n, %	702 (30.0)	780 (33.3)	651 (29.5)	754 (34.2)
Lumbar spine BMD T-score	−2.83 (0.67)	−2.14 (0.80)	−2.84 (0.68)	−2.81 (0.75)
Total hip BMD T-score	−1.85 (0.79)	−1.50 (0.79)	−1.85 (0.79)	−1.93 (0.80)
CTX, ng/mL, median (Q1, Q3)^a^	0.505 (0.357, 0.700)	0.182 (0.086, 0.555)	0.555 (0.420, 0.661)	0.568 (0.426, 0.728)
P1NP, μg/L, median (Q1, Q3)^a^	46.2 (31.5, 56.8)	17.3 (10.3, 26.0)	55.8 (42.5, 65.6)	48.8 (35.0, 67.6)

Abbreviations: n number of subjects enrolled in the extension; Q, quartile. Data are means with SD unless otherwise noted.

a Data are from subjects who were included in the BTM substudy.

### Long-term denosumab group

#### BTMs and BMD

**Figure 2.**
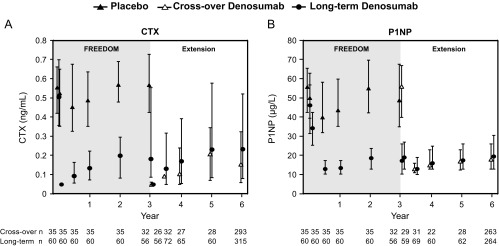
Concentrations of the bone turnover markers serum CTX (A) and serum P1NP (B). Data are medians and interquartile ranges. Time points include the following: baseline, month 1, and years 0.5, 1, 2, 3, 3 (day 10), 3.5, 4, 5, and 6. n, number of subjects with observed data.

Concentrations of serum CTX and P1NP are shown for women in the BTM subset (Figure 2). After the first extension dose (the seventh dose of denosumab), median values for the bone resorption marker CTX were 0.049 ng/mL at day 10 and 0.131 ng/mL at month 6. For the bone formation marker P1NP, median values were 19.0 μg/L at day 10 and 13.0 μg/L at month 6. These results were consistent with those observed after the first dose of denosumab during the parent study. Throughout the 6 years of the continued denosumab treatment, the median BTM values remained below the median values observed at FREEDOM baseline.

**Figure 3.**
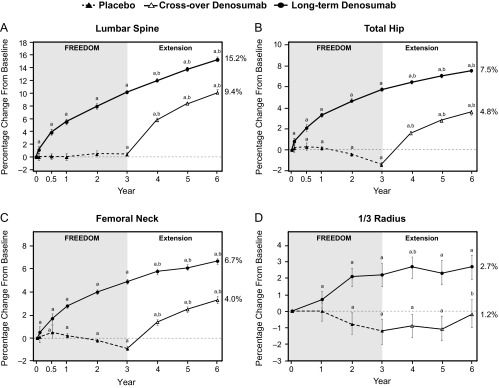
Percentage change from FREEDOM baseline in BMD at the lumbar spine (A), total hip (B), femoral neck (C), and 1/3 radius (D). Data are least squares means and 95% confidence intervals. Data are shown for the entire extension study population for the lumbar spine, total hip, and femoral neck sites. Data from the BMD substudy population are shown for the 1/3 radius. This substudy also provided BMD data for early intermediate time points in the parent trial for the lumbar spine (month 1, month 6, year 1, and year 2), total hip (months 1 and 6), and femoral neck (months 1 and 6). ^a^, *P* < .05 compared with FREEDOM baseline; ^b^, *P* < .05 compared with extension baseline. Percentages listed to the right of each graph represent the percentage change in BMD while on denosumab treatment.

During the first 3 years of the extension, continued increases in BMD occurred with long-term denosumab treatment (4.9% lumbar spine; 1.8% total hip; 1.7% femoral neck; 0.6% 1/3 radius) for cumulative 6-year gains of 15.2% (lumbar spine), 7.5% (total hip), 6.7% (femoral neck), and 2.7% (1/3 radius) (Figure 3).

### Fractures

**Figure 4.**
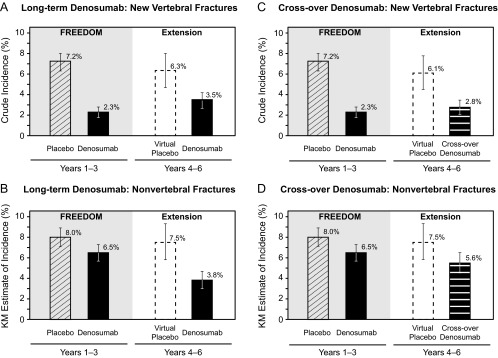
Incidence of new vertebral fractures (A and C) and nonvertebral fractures (B and D). Solid bars represent actual data collected and dashed bars represent virtual placebo data. Percentages for new vertebral fractures are crude incidence and percentages for nonvertebral fractures are Kaplan-Meier estimates. Error bars correspond to 95% confidence intervals.

The incidence of new vertebral fractures during the first 3 years of the extension for the long-term group was 3.5%. Similar rates were obtained for the prespecified PP (3.4%) and MPP (2.9%) subsets. These rates were below those observed in the parent trial placebo group (7.2%) (Figure 4A). They also were below the estimated fracture rates expected had the denosumab participants who enrolled in the extension received placebo for 6 years [virtual placebo; 6.3%; RR 0.56 (0.39–0.78)]. Additionally, the incidence of new and worsening vertebral (3.7%), clinical vertebral (0.6%), and all clinical (4.4%) fractures with long-term denosumab treatment (years 4–6) remained low and below those observed in the parent trial placebo group (7.3%, 2.6%, and 10.2%, respectively; Table 2).

**Table 2.** Fracture Incidence

	Placebo	Denosumab		
	FREEDOM Years 1–3	FREEDOM Years 1–3	Extension Long-Term Years 4–6	Extension Crossover Years 4–6
New and worsening vertebral	7.3%	2.4%	3.7%	3.0%
Clinical vertebral	2.6%	0.8%	0.6%	0.3%
All clinical	10.2%	7.2%	4.4%	5.9%
Major nonvertebral^a^	6.4%	5.2%	2.9%	4.3%
Hip	1.2%	0.7%	0.4%	0.7%

Percentages for new and worsening vertebral fractures are crude incidence. Percentages for clinical vertebral, all clinical, major nonvertebral, and hip fractures are Kaplan-Meier estimates.

a Includes a subset of nonvertebral fractures including pelvis, distal femur (whole femur except proximal), proximal tibia, ribs, proximal humerus, forearm, and hip.

For nonvertebral fractures, the 3-year incidence for the long-term group during the extension was 3.8%. Similar results were obtained for the PP (3.7%) and MPP (3.3%) subsets. These rates were below those observed in the FREEDOM denosumab (6.5%) and placebo (8.0%) groups (Figure 4B). They also were below the estimated fracture rate for the virtual placebo group [7.5%; RR 0.50 (0.36–0.69)]. The incidence rates of major nonvertebral (2.9%) and hip (0.4%) fractures with long-term denosumab treatment (years 4–6) were below those observed in the parent trial placebo group (6.4% and 1.2%, respectively) and the parent trial denosumab group (5.2% and 0.7%, respectively; Table 2). The most common nonvertebral fracture sites in the long-term group during the first 3 years in the extension were wrist (distal ulna and distal radius; n = 31), rib (n = 11), hip (n = 9), and ankle (n = 9) (n is the number of affected women).

### Adverse events

The subject incidence rates per 100 subject-years for all, serious, and fatal AEs in the long-term group during the extension were similar to or lower than those in the placebo and denosumab groups during the parent trial (Table 3). The exposure-adjusted incidence rates of malignancy (1.8 in FREEDOM and 1.9 in the extension period) and infections (29.3 in FREEDOM and 23.4 in the extension period) remained low and showed no trend toward increases over time. The most commonly reported (≥0.1 events per 100 subject-years) malignancy AEs in the long-term group during the extension were basal cell carcinoma, breast cancer, colon cancer, and lung neoplasm (0.5, 0.2, 0.1, and 0.1 events per 100 subject-years, respectively).

**Table 3.** Exposure-Adjusted Subject Incidence of Adverse Events

	Placebo	Denosumab		
	FREEDOM Years 1–3 (N = 3883), Rate, n	FREEDOM Years 1–3 (N = 3879), Rate, n	Extension Long-Term Years 4–6 (N = 2343), Rate, n	Extension Crossover Years 4–6 (N = 2206), Rate, n
All adverse events	156.1 (3614)	154.3 (3598)	106.2 (2067)	104.2 (1944)
Infections	30.7 (2113)	29.3 (2052)	23.4 (1070)	25.0 (1054)
Malignancies	1.6 (167)	1.8 (187)	1.9 (120)	1.8 (108)
Eczema	0.6 (67)	1.1 (119)	1.0 (65)	1.0 (57)
Hypocalcemia	<0.1 (3)	0	<0.1 (1)	<0.1 (6)
Pancreatitis	<0.1 (3)	<0.1 (7)	<0.1 (4^a^)	<0.1 (2)
Serious adverse events	10.4 (974)	10.6 (1002)	10.6 (597)	10.9 (573)
Infections	1.3 (134)	1.5 (160)	1.3 (82)	1.4 (81)
Cellulitis or erysipelas	<0.1 (1)	0.1 (12)	<0.1 (5)	<0.1 (1)
Fatal adverse events	0.8 (90)	0.6 (70)	0.7 (45)	0.7 (41)

Abbreviation: N, number of subjects who received one or more doses of IP; n, total number of subjects with an adverse event. Treatment groups are based on the original randomized treatments received in FREEDOM. All subjects in the extension are receiving denosumab. The rate is the exposure-adjusted subject incidence per 100 subject-years. Adverse events were coded using MedDRA version 13.0.

a Previously reported as 5; one case subsequently was determined to be a coding error.

One midshaft and one subtrochanteric femoral fracture occurred in the long-term group during the first 3 years of the extension. Both were sent for adjudication and neither case was determined to be consistent with atypical femoral fracture.

Four oral events in the long-term group were adjudicated as consistent with ONJ during the first 3 years of the extension. These cases occurred after the participants received their 11th (two participants) or 12th (two participants) doses of denosumab. Two of these participants discontinued denosumab therapy and the other two have continued therapy. All four of the lesions have healed with appropriate treatment.

### Crossover denosumab group

#### BTMs and BMD

Concentrations of serum CTX and P1NP are shown for women in the BTM subset (Figure 2). After the first dose of denosumab in the crossover group, there was a rapid and marked reduction in median serum CTX reaching 0.049 ng/mL at day 10 and 0.092 ng/mL at month 6. For the bone formation marker P1NP, median values were 56.0 μg/L at day 10 and 13.0 μg/L at month 6. These results were consistent with those observed after the first dose of denosumab during the parent study.

The crossover group had significant gains in BMD at the lumbar spine (9.4%), total hip (4.8%), femoral neck (4.0%), and 1/3 radius (1.2%) during the first 3 years of denosumab treatment during the extension, similar to those observed in the long-term denosumab group during the first 3 years of FREEDOM (lumbar spine, 10.1%; total hip, 5.7%; femoral neck, 4.9%; 1/3 radius, 2.2%; Figure 3).

### Fractures

The incidence of new vertebral fractures during the first 3 years of denosumab treatment in the extension for the crossover group was 2.8%. Similar results were obtained for the prespecified PP (2.9%) and MPP (2.9%) subsets. These rates were similar to the 2.3% observed in the 3-year parent trial for the group that received denosumab and well below the 7.2% observed in the parent trial placebo group (Figure 4C). They also were below the estimated fracture rate for the virtual placebo group [6.1%; RR 0.46 (0.31–0.67)]. The incidence of new and worsening vertebral (3.0%), clinical vertebral (0.3%), and all clinical (5.9%) fractures with crossover denosumab treatment (years 4–6) remained low and below those observed in the parent trial placebo group (7.3%, 2.6%, and 10.2%, respectively; Table 2).

For nonvertebral fractures, the 3-year incidence for the crossover group during the extension was 5.6%. Similar results were obtained for the prespecified PP (5.4%) and MPP (5.4%) subsets. These rates compare favorably with those observed in the FREEDOM denosumab (6.5%) and placebo (8.0%) groups (Figure 4D). They also were below the estimated fracture rate for the virtual placebo group [7.5%; RR 0.74 (0.54–0.99)]. The incidence of major nonvertebral (4.3%) and hip (0.7%) fractures with crossover denosumab treatment (years 4–6) remained low and below those observed in the parent trial placebo group (6.4% and 1.2%, respectively; Table 2). The most common nonvertebral fracture sites in the crossover group during the first 3 years in the extension were wrist (distal ulna and distal radius; n = 49), ankle (n = 15), hip (n = 14), humerus (n = 9), and foot (n = 9).

### Adverse events

During the first 3 years of the extension period, the subject incidence rates of all, serious, and fatal AEs in the crossover group were similar to or lower than those reported by the FREEDOM placebo and denosumab groups (Table 3). For example, the exposure-adjusted incidence rates of malignancy were 1.8 in FREEDOM and 1.8 in the extension, and for infection, they were 29.3 in FREEDOM and 25.0 in the extension. The most commonly reported (≥0.1 events per 100 subject-years) malignancy adverse events in the crossover group during the extension were basal cell carcinoma and breast cancer (0.4 and 0.2 events per 100 subject-years, respectively).

There were two midshaft femur fractures in the crossover group during the first 3 years of the extension. Both were sent for adjudication and one was considered by the panel to be consistent with atypical femoral fracture. It occurred after the participant had received six doses of denosumab. The fracture was treated by internal fixation and a 12-week, postoperation x-ray showed healing and callus. The participant has discontinued therapy.

During the first 3 years of the extension period, two oral events in the crossover group were adjudicated as consistent with ONJ. These cases occurred after the participants received their third and fourth dose of denosumab, respectively. One participant discontinued therapy and the other continued to receive denosumab without further oral events. Both lesions have healed with appropriate treatment.

## Discussion

In the FREEDOM trial, denosumab was shown to increase BMD, reduce BTMs, and decrease the risk of new vertebral and nonvertebral fractures (8). The purposes of the study extension are to evaluate the long-term safety and efficacy of continued denosumab administration (long- term group) in a large number of participants and to assess the consistency of findings from 3 years of denosumab treatment (crossover group) as compared with the parent study.

For the long-term group that continued denosumab for a total of 6 years, BTMs were maintained at lower than pretreatment levels and BMD continued to increase. These distinctive long-term effects on BMD confirm the results of the extended phase 2 study (9) and are consistent with the previously reported effects of denosumab on trabecular and cortical bone structure (15–17). Evidently the apparent differences from bisphosphonate effects reflect the differences in biological mechanisms. However, exactly how the differences in drug mechanism of action result in the observed differences in structural effects remains to be fully elucidated. Fracture incidence in the long-term group remained low and below the rates reported in the FREEDOM placebo group. New vertebral and nonvertebral fracture rates were also lower than the fracture rates that would have been expected if extension participants had received placebo, as estimated with a previously reported methodology (14). As discussed previously (10), this methodology is improved from a previous osteoporosis study (18), not only in the sophistication of the model but also in the use of data from the placebo group of the same trial rather than a different population. These observations are consistent with a 6-year maintenance of effect by denosumab to reduce fractures.

A main objective of the extension was to further characterize the safety of denosumab. Importantly, the subject incidence rates of adverse events reported by participants in the long-term group during the first 3 years of the extension were similar to or lower than those reported by the FREEDOM placebo and denosumab groups. There was no evidence of increased frequency of any specific event, including malignancy and infections (including erysipelas and cellulitis), despite the fact that the participants were aging. Cases consistent with atypical femoral fracture and ONJ have been observed in patients receiving denosumab in this study, but the incidence remains low.

Because the cross-over group was treated with denosumab for 3 years during the extension, there was a unique opportunity to compare these efficacy and safety results with those obtained during the 3 years of denosumab treatment in the parent trial. The data obtained were consistent with FREEDOM observations: rapid and marked reduction in BTMs, large increases in BMD, low fracture rates, and a favorable benefit/risk profile. These data provide further supportive evidence for the consistency of results obtained with denosumab treatment.

The ultimate objective of osteoporosis treatment is the reduction of fractures. Although this study did not have a long-term placebo group, it is particularly noteworthy that the rates of all fractures during the first 3 years of the extension, for both the long-term and crossover denosumab groups, were similar to or lower than the 3-year rates observed during the parent trial for the denosumab group. Interestingly, the rate of new vertebral fractures in the crossover group was similar to that observed in the extension long-term group. For nonvertebral fractures, the rate in the crossover group was similar to that seen in the denosumab group during the parent trial, whereas the rate in the long-term group appears to have declined further. The comparisons with the virtual placebo model are consistent with these findings.

It was recently reported that gains in total hip BMD predict a considerable proportion of the fracture risk reduction observed with denosumab in the parent trial (15), suggesting the possibility of using change in total hip BMD as a predictor for the effect of denosumab treatment on fracture risk. With this in mind, the continued gains in total hip BMD observed over 6 years of treatment with denosumab in this extension study are encouraging.

One inherent limitation of the study is that not all qualified FREEDOM participants enrolled in the extension, so long-term efficacy and safety observations were limited to those who were eligible and chose to participate. In addition, the open-label design of the study and the lack of a continuing placebo group precluded direct comparisons of results from denosumab-treated participants and placebo-treated participants for all measures. A virtual placebo (virtual twin) model was used to estimate the fracture risk for a placebo-treated population otherwise similar to the denosumab-treated subjects.

In summary, in the ongoing extension to the FREEDOM trial, 6 years of denosumab treatment of postmenopausal women with osteoporosis was associated with sustained but not progressive decreases in bone turnover, continued increases in BMD, and maintenance of low fracture rates. Efficacy data from the cross-over group were consistent with observations from the 3 years of the original FREEDOM trial. There was no apparent increase in the risk of AEs with extended exposure or with cross-over from the placebo group.
